# Gender Differences in Immune Reconstitution: A Multicentric Cohort Analysis in Sub-Saharan Africa

**DOI:** 10.1371/journal.pone.0031078

**Published:** 2012-02-17

**Authors:** David Maman, Mar Pujades-Rodriguez, Fabien Subtil, Loretxu Pinoges, Megan McGuire, Rene Ecochard, Jean-François Etard

**Affiliations:** 1 Epicentre, Médecins Sans Frontières, Paris, France; 2 Hospices Civils de Lyon, Service de Biostatistique, Lyon, France; 3 TransVIHMI, UMI 233, Montpellier, France; 4 Université de Lyon, Lyon, France; 5 Université Lyon I, Villeurbanne, France; 6 CNRS UMR5558, Laboratoire de Biométrie et Biologie Evolutive, Equipe Biostatistique Santé, Pierre-Bénite, France; University of Cape Town, South Africa

## Abstract

**Background:**

In sub-Saharan Africa, men living with HIV often start ART at more advanced stages of disease and have higher early mortality than women. We investigated gender difference in long-term immune reconstitution.

**Methods/Principal Findings:**

Antiretroviral-naïve adults who received ART for at least 9 months in four HIV programs in sub-Saharan Africa were included. Multivariate mixed linear models were used to examine gender differences in immune reconstitution on first line ART.

A total of 21,708 patients (68% women) contributed to 61,912 person-years of follow-up. At ART start,. Median CD4 at ART were 149 [IQR 85–206] for women and 125 cells/µL [IQR 63–187] for men. After the first year on ART, immune recovery was higher in women than in men, and gender-based differences increased by 20 CD4 cells/µL per year on average (95% CI 16–23; *P*<0.001). Up to 6 years after ART start, patients with low initial CD4 levels experienced similar gains compared to patients with high initial levels, including those with CD4>250cells/µL (difference between patients with <50 cells/µL and those with >250 was 284 cells/µL; 95% CI 272–296; LR test for interaction with time *p* = 0.63). Among patients with initial CD4 count of 150–200 cells/µL, women reached 500 CD4 cells after 2.4 years on ART (95% CI 2.4–2.5) and men after 4.5 years (95% CI 4.1–4.8) of ART use.

**Conclusion:**

Women achieved better long-term immune response to ART, reaching CD4 level associated with lower risks of AIDS related morbidity and mortality quicker than men.

## Introduction

According to UNAIDS, more than 3.9 million patients were receiving antiretroviral therapy (ART) in sub-Saharan Africa at the end of 2009. This represents 37% of those in need of treatment and an increase of one million patients in one year [1]. Early initiation of ART in the course of disease is associated with better survival [2], [3] and better long-term immune reconstitution [4], [5]. Indeed, in 2009, the World Health Organization (WHO) raised the recommended CD4 threshold for ART initiation to 350 cells/µL [1], [6].

Immune recovery after ART initiation follows two phases: a rapid increase during the first months, followed by a slower one thereafter [7], [8], [9]. They correspond to different biological mechanisms: the first phase of rapid CD4 cell increase is linked to the relapse of pre-trapped CD4 cells [10], while the second phase corresponds to a proliferation of new CD4 cells, related to thymus reactivation, which progressively reconstitutes stocks of CD4 cells [11]. The feasibility of complete reconstitution remains unknown, as do influencing factors.

Most of large cohort studies describing long-term immune reconstitution have been performed in North American cohorts [12], [13]. In sub-Saharan Africa, the gender differences at ART start are important. In these settings, more women than men are infected [14], [15], as the majority of infections happen through heterosexual transmission. Furthermore, men are frequently underrepresented in ART cohorts, and they initiate ART at a more advanced stage of the disease with a lower CD4 cell counts. [16], [17], [18] In addition, they experience higher early mortality compared with women [17], [19], [20], [21].

This study explored gender differences in immune reconstitution after the first 9 months of ART, to focus on the second phase of immune reconstitution, in four sub-Saharan African HIV programs supported by Médecins Sans Frontières (MSF).

## Methods

### Data Analysis and Study Population

In collaboration with national Ministries of Health, MSF began providing free HIV care and antiretroviral therapy (ART) in four programs in Malawi, Uganda, and Kenya between March 2001 and July 2002. A standardized surveillance system was implemented to collect routine demographic, clinical, and treatment data (FUCHIA software, Epicentre, Paris, France). Data consistency checks were implemented to ensure the data quality.

The databases used for the analysis were locked on 30 November 2009. ART initiation was based on 2003 and 2006 WHO recommendations. Patients received non-nucleoside reverse transcriptase inhibitor-based first-line therapy in the form of fixed-dose combinations. Adherence support was provided by trained counsellors prior to ART start and during follow-up on treatment. CD4 cell counts were measured every 6 or 12 months, depending on the program, initially using manual methods (Dynabeads, Dynal Biotech SA, Compiègne, France), and since mid-2003 with semi-automated techniques (Cyflow counter, Partec, Münster, Germany).

**Figure 1:**
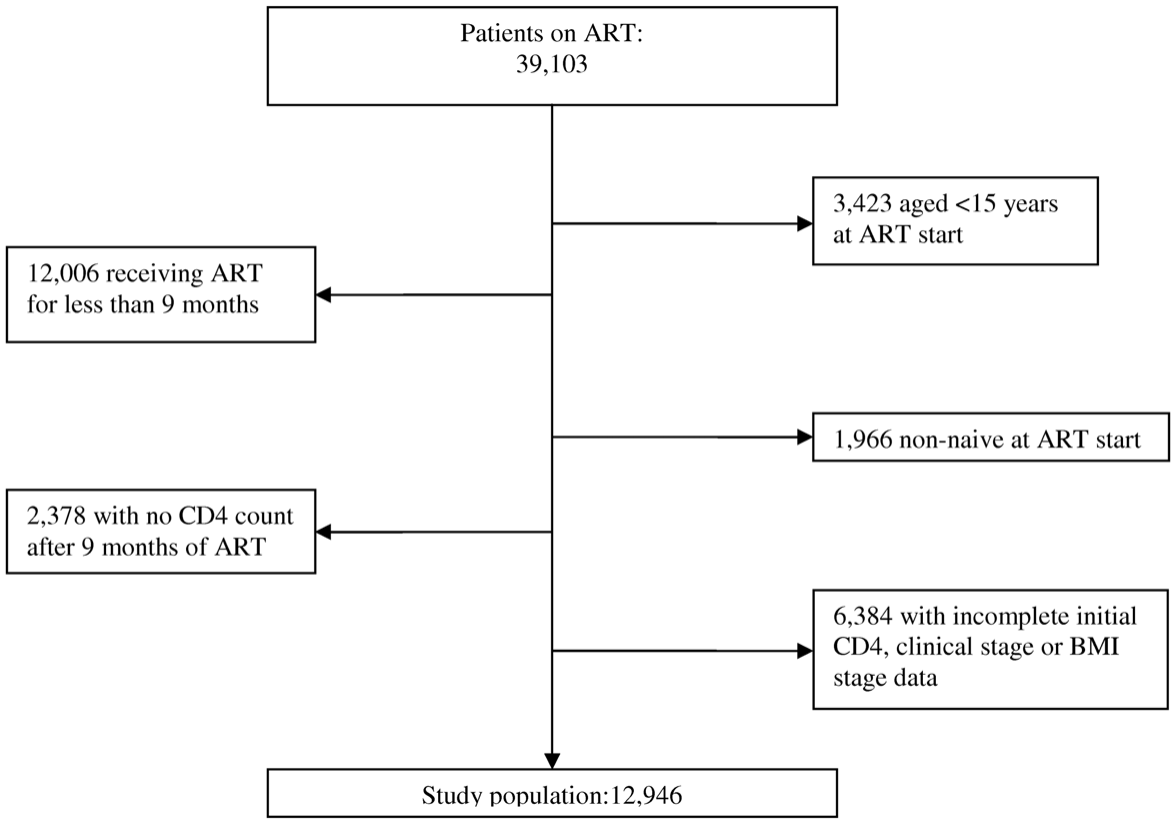
Patient data inclusion flow chart.

Patients were included in the study if they (a) had initiated ART in one of the MSF-supported programs, (b) were aged 15 years or older, (c) were ART-naïve at the time of therapy start, (d) received ART for at least 9 months (Figure 1), (e) had complete information about baseline covariates and (f) had at least one CD4 count after 9 months of ART. We included patients at 9 months after ART start because we wanted all the CD4 measures taken at one year to be included, which can sometimes be taken a few months before or after; thus the 9th month was chosen as the inclusion benchmark.

The last CD4 measurement considered was the one collected at end of follow-up, at switch to second-line therapy, at transfer out from the program, or closest to time of death, whichever occurred first.

### Study Definitions and Statistical Methods

Loss to follow-up was defined as the absence of a clinical visit for at least 9 months (among patients who did not die or were not transferred out of the program) as patients had up to 6 months between two follow-up appointments. Second-line therapy was defined as a protease inhibitor-containing regimen associated with a concomitant change of at least one nucleoside reverse transcriptase inhibitor, as described previously in other studies [22].

For each patient, the initial CD4 cell count was the measurement taken between 3 months before and 1 month after ART start. If more than one measurement was taken, the closest to ART start was considered.

CD4 cell counts were described using medians and interquartile ranges (IQR). CD4 response to ART was the main outcome variable. Random intercept and coefficient mixed linear models with a second-degree natural polynomial of time [23], [24] were used to model long-term immune reconstitution and investigate differences by gender. Random effects characterize between-subject variations whereas fixed effects represent the mean value of a coefficient within the cohort. These models take into account the correlation between CD4 measures within each individual.

Factors considered in adjusted models were time on ART, initial CD4 categories (<50, 50–99, 100–149, 150–199, 200–249, ≥250 cells/µL), body mass index (BMI) (<16, 16–16.99, 17–17.99, ≥18 kg/m^2^) at ART start, WHO clinical stage at ART start (stages 1 to 4), age at ART start (<30, 30–50, >50 years old), and HIV program. Likelihood ratio (LR) tests and Wald tests for association were used with a significance level of 0.05.

Predicted evolution of mean CD4 counts over time since ART start were plotted, stratified by initial CD4 levels and gender. For female and male patients included in each CD4 cell strata, the mean time to reach a threshold of 500 cells/µL was predicted. The corresponding 95% CI were estimated using the delta method [25]. Data were analyzed using Stata 11 (Stata Corp., College Station, Texas, USA).

This multicentre study was based on analysis of routinely collected, patient monitoring data from the programmes that Médecins Sans Frontières is running in partnership with the local ministry of health for more than 10 years. The data were de-identified before we accessed them. Verbal consent for use of monitoring data is routinely obtained from patients at first consultation, The study protocol and the consent procedure were approved by the Comité de Protection des Personnes d'Ile de France (Ref 10,032), Paris, France.

## Results

### Patient Characteristics at ART Initiation

Of 39,103 patients who ever received ART in the four HIV programs, 12,946 met the inclusion criteria (Figure 1) and contributed 33,622 person-years at risk to the analysis. Patient median follow-up time since ART start were 2.77 years [IQR 1.86–4.17] for women and 2.64 [IQR 1.74–3.91] for men.

Patients treated in the Malawian cohort represented 52% of eligible patients (Table 1). Women accounted for 69% of patients. At ART start, median age was 36 years [IQR 30; 42]. Among them, 19% of women and 24% of men were classified WHO clinical stage 4. Median initial CD4 count was 141 cells/µL [IQR 77–200], and were higher in women [149 cells/µL; IQR 85–206] than in men [125 cells/µL; IQR 63–187].

**Table 1:** Study population characteristics at ART start, Uganda, Kenya, and Malawi, 2001–2009.

	Women (%)	Men (%)	Total (%)
	N = 8,878	N = 4,068	N = 12,946
**HIV program**			
Homa-Bay, Kenya	1,320 (15.1)	616 (15.1)	1,936 (15)
Mathare, Kenya	861 (9.4)	384 (9.4)	1,245 (10)
Chiradzulu, Malawi	4,501(48.9)	1,989 (48.9)	6,490 (50)
Arua, Uganda	2,196 (26.5)	1,079 (26.5)	3,275 (25)
**Age (years)**			
>50	666 (7.5)	603(14.8)	1,269 (10)
30–50	5,469 (61.6)	2,963 (72.8)	8,432 (65)
<30	2,743 (30.9)	502 (12.3)	3,245 (25)
**Clinical stage**			
Stage 1	1,369 (15.4)	497 (12.2)	1,866 (14)
Stage 2	2,006 (22.6)	770 (18.9)	2,776 (21)
Stage 3	3,832 (43.2)	1,833 (43.2)	5,665 (44)
Stage 4	1,671 (18.8)	968 (23.8)	2,639 (20)
**BMI (kg/m^2^)**			
<16	482 (5.4)	217 (5.3)	699 (5)
16–16.99	524 (5.9)	259 (6.4)	2,776 (6)
17–17.99	807 (9.1)	532 (13.1)	5,665 (10)
≥18	7,065 (79.6)	3,060 (75.2)	10,125 (78)
**CD4 (cells/µL)**			
<50	1,166 (13.1)	812 (20.0)	1,978 (15)
50–99	1,548 (17.4)	826 (20.3)	2,374 (18)
100–149	1,728 (19.5)	781 (19.2)	2,509 (19)
150–199	1,985 (22.4)	838 (20.6)	2,823 (22)
200–249	1,540 (17.4)	572 (14.1)	2,112 (16)
≧250	911 (10.3)	239 (5.9)	1,150 (9)

BMI, body mass index.

At ART start, median BMI for women and men were 20.2 kg/m^2^ [IQR 18.4–22.3] and 19.5 kg/m^2^ [IQR 18.0–21.1], respectively.

At the end of follow-up, 8,878 women (91%) and 3,638 men (89%) patients were alive and receiving care, 206 men (5%) and 349 women (4%) had been lost to follow-up, 80 men (2%) and 229 women (3%) transferred to other programs. Among the study population 84 men (2%) and 119 women (1%) had died, and 60 men (1%) and 129 women (1%) had initiated second-line treatment.

### Observed CD4 Response in Women and Men

**Figure 2:**
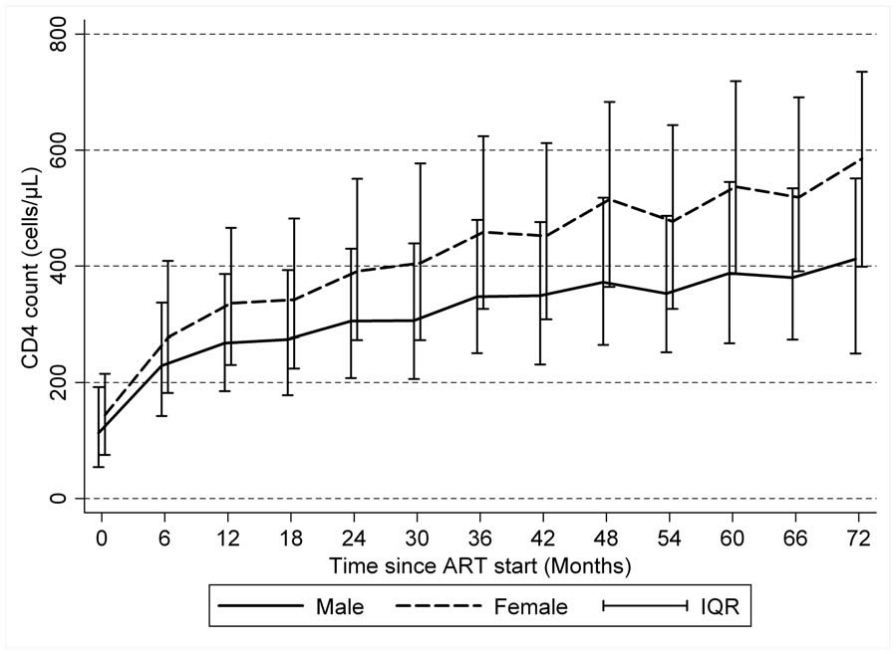
Observed median CD4 cell counts after ART start, by gender, Uganda, Kenya, and Malawi, 2001–2009. N = 12,946 patients, 33,622 person-years.

Median CD4 cell counts increased with time on ART (Figure 2). The difference between men and women CD4 response also increased with time on ART. At 1 year of ART, women had a median CD4 count of 336 cells/µL [IQR 230–466], compared with 268 cells/µL [IQR 185–387] in men. At 6 years of ART, median CD4 cell count for women and men were 585 cells/µL [IQR 399–735] and 412 cells/µL [IQR 250–551], respectively. Among those who initiated ART with initial CD4 cell count below 50 cells/µL, 134 (15%) women and 127 (19%) men failed to reach a CD4 count of 100 cells/µL after one year on ART.

### Modelling Long-Term CD4 Response in Women and Men

Detailed results of the model to explore the association between gender and CD4 recovery among patients followed up for more than 9 months on ART are presented in Table 2. From the 21,708 patients meeting the inclusion criteria, 12,946 patients with complete information on all the covariates at baseline and at least one CD4 measure after 9 months on ART contributed to the analysis.

**Table 2:** Associations between gender and other factors and mean CD4 response to ART.

	N (%) Total: 12,946	Adjusted difference in mean CD4 in cells/µL (95% CI)	Adjusted mean CD4 change per year in cells/µL (95% CI)	*P*-value
**Time (year)**			65 (60–69)	<0.001*
**Time squared**			−6 (−6, −5)	<0.001*
**Gender×time interaction (female vs male)**			20 (16–23)	<0.001*
**Gender**				<0.001**
Male	4,068 (31)	Reference‡		
Female	8,878 (69)	40 (34–46)		
**Programme**				<0.001**
Homa-Bay, Kenya	1,936 (15)	Reference‡		
Mathare, Kenya	1,245 (10)	−72 (−83, −60)		
Chiradzulu, Malawi	6,490 (50)	−31 (−39, −22)		
Arua, Uganda	3,275 (25)	−52 (−60, −42)		
**Age (years)**				<0.001**
>50	1,269 (10)	Reference‡		
30–50	8,432 (65)	21 (12–30)		
<30	3,245 (25)	52 (42–62)		
**Initial clinical stage**				0.04**
Stage 1	1,866 (14)	Reference‡		
Stage 2	2,776 (21)	−11 (−20, −2)		
Stage 3	5,665 (44)	−12 (−21, −4)		
Stage 4	2,639 (20)	−11 (−20, −2)		
**Initial BMI (kg/m^2^)**				0.20**
<16	699 (5)	Reference‡		
16–16.99	2,776 (6)	−14 (−30, +1)		
17–17.99	5,665 (10)	−15 (−29, −1)		
≥18	10,125 (78)	−12 (−24, 1)		
**Initial CD4 count (cells/µl)**				<0.001**
<50	1,978 (15)	Reference‡		
50–99	2,374 (18)	46 (36–55)		
100–149	2,509 (19)	107 (98–117)		
150–199	2,823 (22)	167 (157–176)		
200–249	2,112 (16)	207 (197–217)		
≧250	1,150 (9)	284 (272–296)		

*P value from Wald test for association model adjusted for all variables included in the table.

**P value from likelihood ratio test for association from the multivariable linear mixed model adjusted for all variables included in the table.

‡ 214 cells/µL (95% CI 195–233) represents the mean CD4 count after 1 year of ART predicted by the multivariable linear mixed model for the reference level of all the variables included in the table.

BMI, body mass index.

CD4 count increased regularly during the first 6 years after ART start, with a progressive slowing of immune reconstitution with time on ART.

Women experienced better reconstitution than men, after accounting for all the variables included in the model. After 1 year on ART, women had CD4 count 40 cells/µL (95% CI 34–46) higher than men, with a yearly average increase of 20 CD4 cells/µL higher than men (95% CI 16–23, *P*<0.0001).

Intervals between initial CD4 strata remained constant over time, as there was no interaction between initial CD4 and time on ART (LR test for interaction with time, *P* = 0.63). Patients with higher initial CD4 count reached higher CD4 levels after ART start. For example, 1 year after ART start, patients who initiated ART with CD4 count 100–150 cells/µL had a CD4 count on average 107 cells/µL higher than patients with initial count <50 cells/µL (95% CI 98–117). This difference increased with higher initial CD4 levels and was 284 cells/µL (95% CI 272–296) for patients with initial CD4 of 250 cells/µL or more compared to those with <50 cells/µL (LR test for trend, *P*<0.001).

Age was also associated with immune reconstitution. Patients younger than 30 years old when they initiated ART had mean CD4 count 52 cells/µL (95% CI 42–62) higher than those aged more than 50 years, one year after ART start (LR test, *P*<0.001). Associations with initial BMI and WHO clinical stage were less strong and did not reach statistical significance when the other variables were accounted in the model.

**Figure 3:**
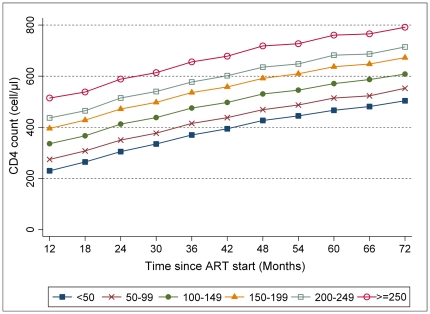
Predicted female mean CD4 count after ART start by initial CD4 level, Uganda, Kenya, and Malawi. N = 8,878; mixed multivariate polynomial model.

**Figure 4:**
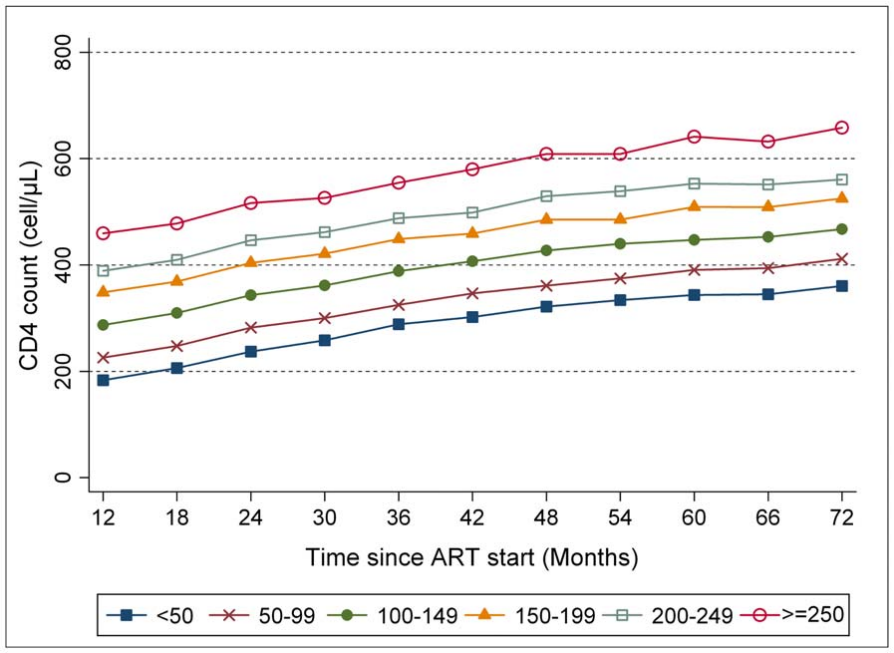
Predicted male mean CD4 count after ART start by initial CD4 level, Uganda, Kenya, and Malawi. N = 4,068; mixed multivariate polynomial model.

After 3 years of ART, women and men who had initial CD4 count 100–149 cells/µL reached 475 cells/µL (95% CI 468–482) and 388 cells/µL (95% CI 379–397), respectively (Figures 3 & 4). After 6 years of ART, the same group of patients reached 608 cells/µL (95% CI 594–621) and 467 cells/µL (95% CI 449–484), respectively. After the first year of ART, the higher the initial CD4 cell count, the higher the level of CD4 count was achieved, and these differences continued over time.

For predicted mean time from ART start to reach CD4 500 cells/µL (Table 3), women with initial count <50 cells/µL needed 5.8 years (95% CI 5.5–6.1), while men with the same initial CD4 count were unable to attain this CD4 level. Those men with pre-treatment CD4 count 200–249 cells/µL reached this threshold at 3.2 years (95% CI 3.0–3.4) after ART start whereas it would take only 1.8 years (1.7–1.9) to women.

**Table 3:** Time in years from ART start to reach CD4 count 500 cells/µL.

	Women (95% CI)	Men (95% CI)
**Initial CD4 count (cells/µL)**		
<50	5.8 (5.5–6.1)	NA
50–99	4.6 (4.5–4.7)	NA
100–149	3.4 (3.3–3.5)	NA
150–199	2.4 (2.3–2.5)	4.5 (4.1–4.8)
200–249	1.8 (1.7–1.9)	3.2 (3.0–3.4)
≧250	Before one year	1.6 (1.5–1.7)

Note: 95% confidence interval obtained using the Delta method.

## Discussion

In this multisite analysis of four large sub-Saharan African HIV programs, long-term immune response up to 6 years after ART start was higher in women compared with men,, independently of characteristics at initiation.

The gender-based difference in immune reconstitution described here has inconsistently been found in previous studies in resource-rich countries, some studies showing a better female immune reconstitution [26], [27] whereas two literature reviews indicated that most of the studies did not find any viroimmunological difference between men and women [28], [29].

In resource-limited settings, studies showing worse male reconstitution are more common. In Cameroon and in China, two studies recently showed male poorer immune reconstitution even after initial patients characteristics were accounted for in the analysis. [30], [31]. In another large multicentric study in sub-Saharan Africa, a gender difference in immune reconstitution was found, but the difference was explained by baseline characteristics [32]. In Senegal, a cohort study found a borderline gender effect which disappeared when viral load and baseline CD4 levels were integrated in the model. [4] At last, a recent cohort study in Tanzania found gender differences in mortality, rates of loss of follow-up or side effects in men but failed to find a significant difference in immune reconstitution after 3 months of ART [33].

Our study confirmed that gender differences in immune reconstitution remained even when baseline factors are included the model. We also showed that this difference increased with time on ART up to 6 years after initiation. Furthermore, we quantified this difference to show that after 6 years on ART, it reached 140 CD4 cells/µL. When we estimated the time on ART required to reach 500 CD4 cells/µL, we found that men remained much longer under this threshold, known to be associated with higher morbidity [2], [34] and mortality [34].

Behavioural, biological factors or a combination of both could explain this difference. First, it is known that men living with HIV usually initiate ART at more advanced stages of disease, resulting in higher mortality [19], [20], [21] highlighting gender differences in health seeking behaviours. Therefore, one possible reason for this gender difference in immune reconstitution could also be behavioural and related to a poorer adherence of men to ART [31], [32]. Decentralization of HIV care in sub-Saharan Africa has been implemented through primary health facilities, and traditionally such care has been well attended by women and children but less so by men. [35] Interventions specifically addressing men's needs might be effective to improve treatment outcomes in this patient population. However, we could not verify this hypothesis as adherence and viral load were not routinely monitored in our cohorts.

Nevertheless, another explanation needs to be explored, reflecting a possible biological explanation. It is known that women have higher physiological CD4 levels compared to men [35], [36], [37] and some studies also underlined the effect of male hormones on the thymic function [37]. Therefore, this gender difference in immune reconstitution could also be linked to a higher potential for women to regenerate their CD4 lymphocytes stock on ART.

We lack of information on women previously exposed to “prevention to mother to child transmission” treatment. Previous exposure to ART (like single dose nevirapine) might underestimate the difference between men and women. The underlining hypothesis would be that women who took these prophylaxis would be more likely to be resistant to ART and thus to have a non optimal immune response. However, recent evidences seem to minimise this influence. [36]

Gender differences in long-term survival outcomes need to be explored and linked to this observed difference in immune reconstitution.

CD4 gains up to 6 years after ART start were similar regardless of the initial CD4 level. We demonstrated for the first time in sub-Saharan African countries that patients who started ART with CD4 count over 250 cells/µL achieved similar CD4 gains up to 6 years after ART initiation than those who initiated at lower CD4 levels. If the influence of initial CD4 level on future reconstitution was well known [4], [8], [12], [13], [38], [39], such levels of reconstitution for patients who initiated ART at high CD4 levels had never been observed previously in sub-Saharan settings. This finding supports the theory that patients may have the same reconstitution potential regardless of baseline CD4. We need to evaluate if complete reconstitution could be feasible if patients were initiated ART at even earlier stage.

This study suggests that long-term CD4 recovery is independent of the level of CD4 cells at ART start, and patients who initiated ART at earlier stages of disease spend shorter time below the threshold of 500 CD4 cells/µL, which is associated with high morbidity [40] and mortality [41], [42]. An earlier start of ART would decrease this high-risk period and facilitate the provision of care in areas suffering from human resources shortages.

Consistent with previous studies, advanced age at initiation was associated with poorer immune reconstitution [27], [43]. This might relate to greater difficulties to reactivate the thymic function and/or to poorer sustained adherence achieved by older patients [44].

We decided to study patient immune response after the first 9 months of ART because during the early stages of treatment, the release of pre-trapped CD4 cells could influence the observed response [45]. The large number of patients included in this analysis allowed careful investigation and quantification of the dose-response effect of initial CD4 counts on immune reconstitution. Indeed, this study used data from four large operational HIV programs running for almost a decade, giving strength to our findings.

Nevertheless, given the definition of inclusion criteria, exclusion of patients because of incomplete initial characteristics is likely to have overestimated the extent of the overall immune reconstitution. Indeed, patients with advanced HIV disease have often been prescribed ART without having initial CD4 count data. Since these patients were likely to have low initial CD4 count values, missing values on initial CD4 count did not follow a random process. Imputation of these missing values was therefore not recommended. Cohort attrition might also have biased estimates. Patients who were included in the analysis were those who survived the first months of ART when mortality is higher. We analyzed routinely collected data without double entry, which could not be implemented for cohorts of this size, though consistency checklists were routinely used to try to reduce data entry errors.

Concerning the generalization of these findings, MSF support on these programs needs to be accounted, as these programs might differ from regular Ministry of Health programs.

In this analysis, we found that women treated for HIV experienced higher immune reconstitution than men, and patients who survived after the first 9 months on ART. Reasons for the gender effect should primarily be investigated through differences in health-seeking and adherence behaviour and addressed to improve outcomes among men.

The paper has not been published or submitted for publication elsewhere. This study was presented at the Conference on Retrovirus and Opportunistic Infections CROI in Boston (David Maman, Mar Pujades-Rodriguez, Fabien Subtil, Loretxu Pinoges, Megan McGuire, René Ecochard, Jean-François Etard. Gender Differences in Long-term Immune Response to ART and Mortality: A Cohort Analysis in 4 Sub-Saharan HIV Programs, CROI Conference, Boston, USA, February 27^th^–March 2^nd^ 2011.).
